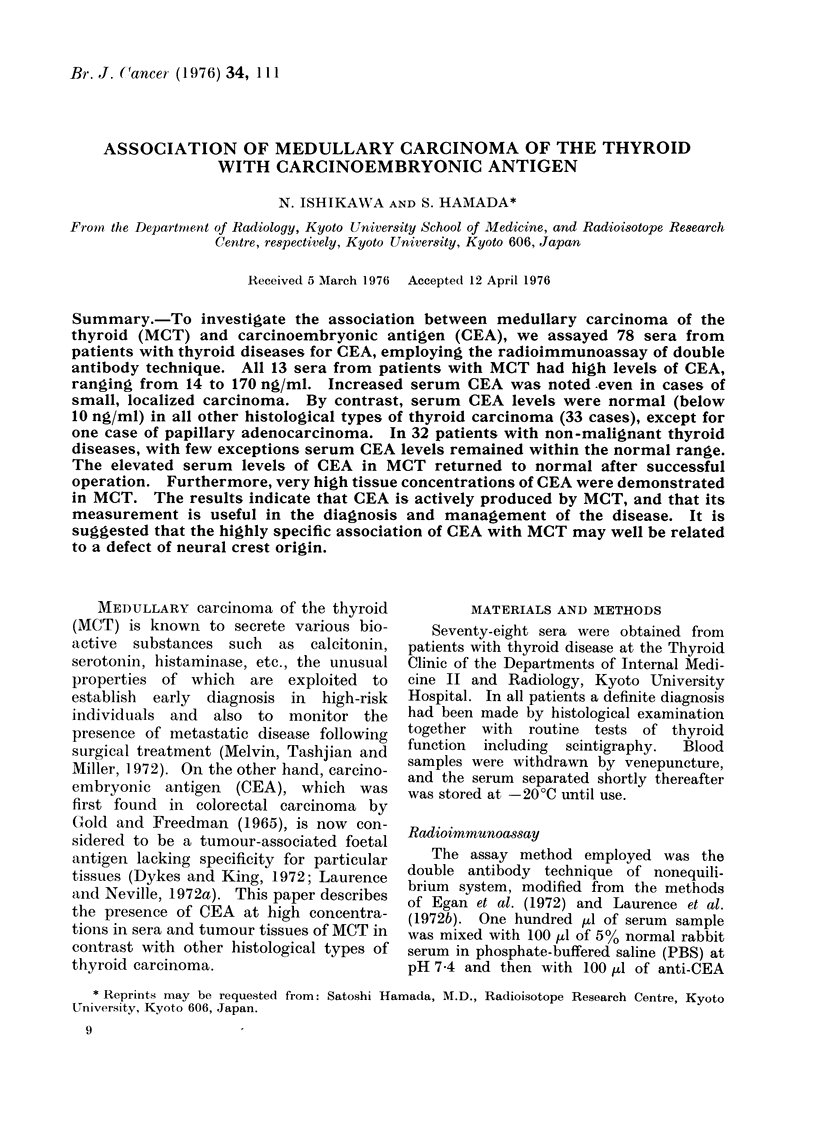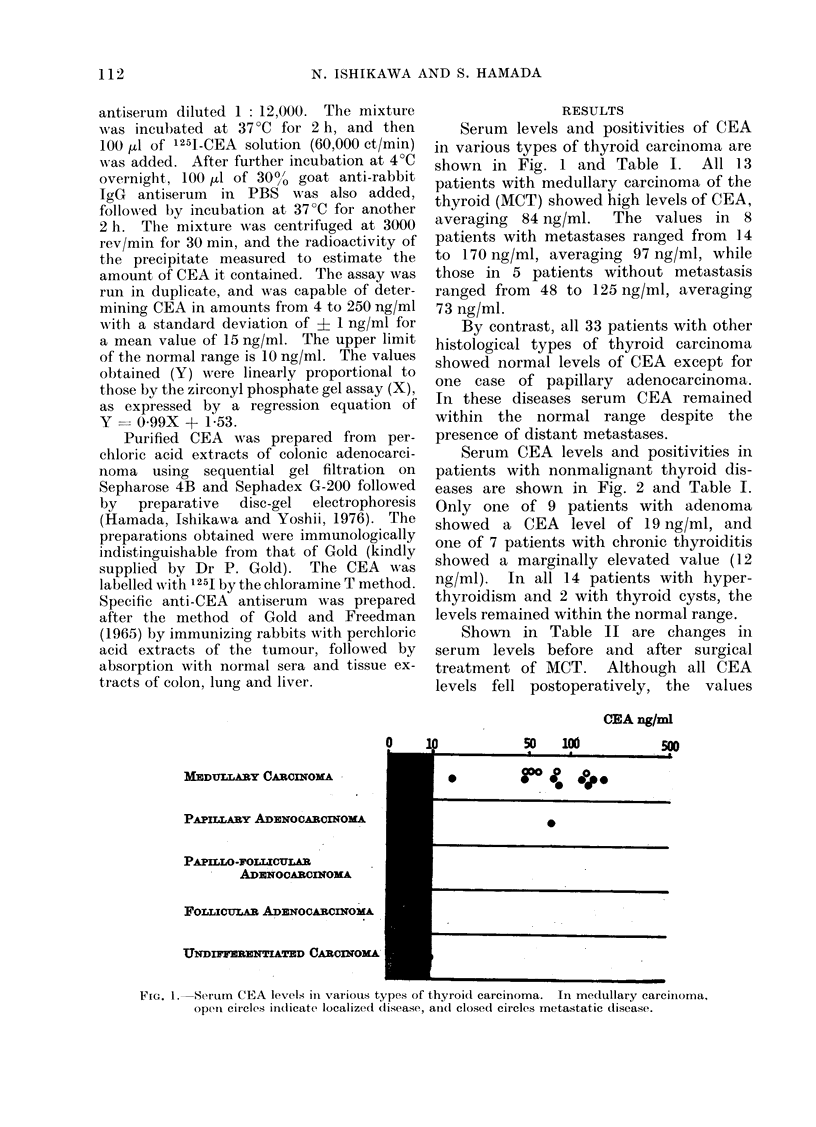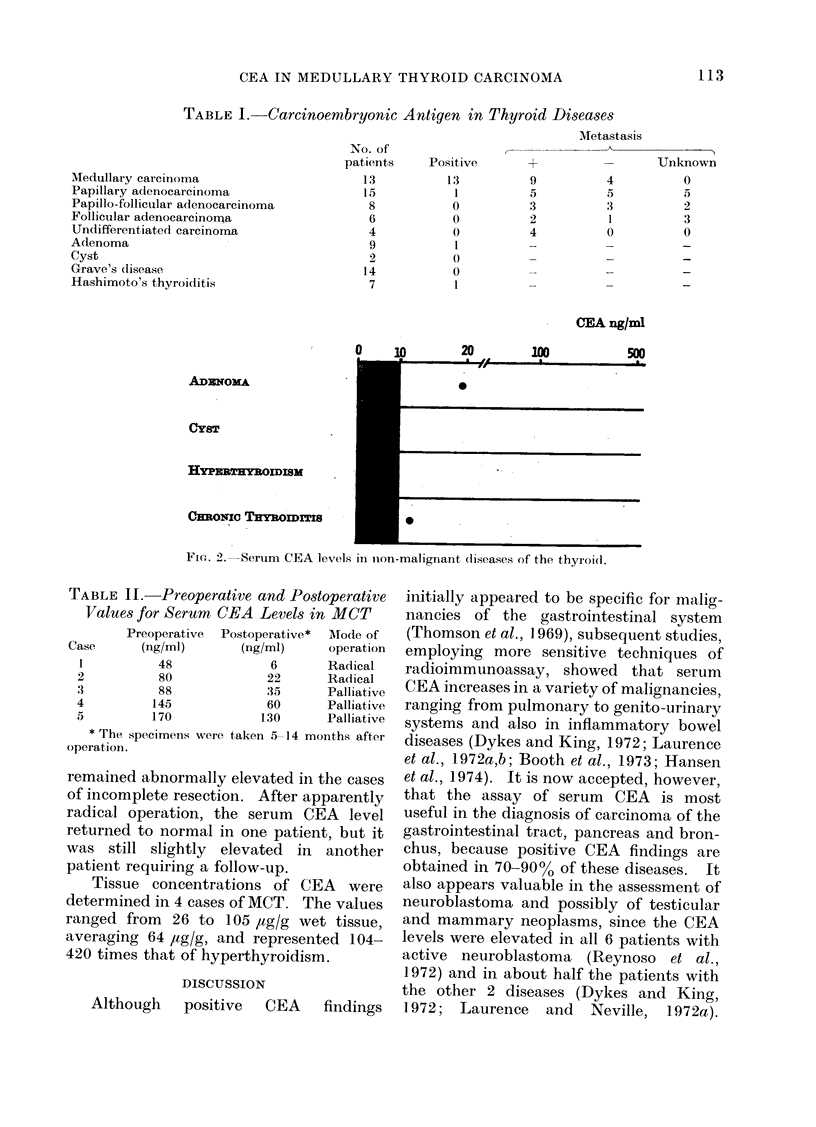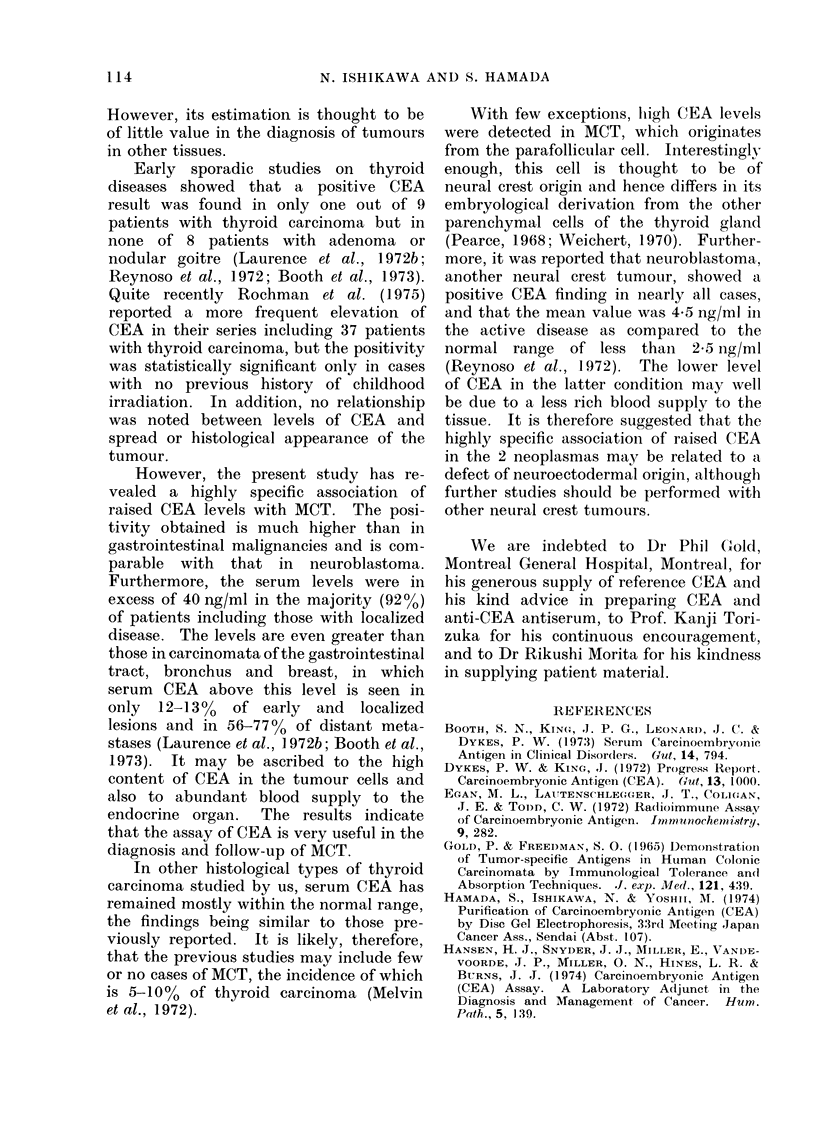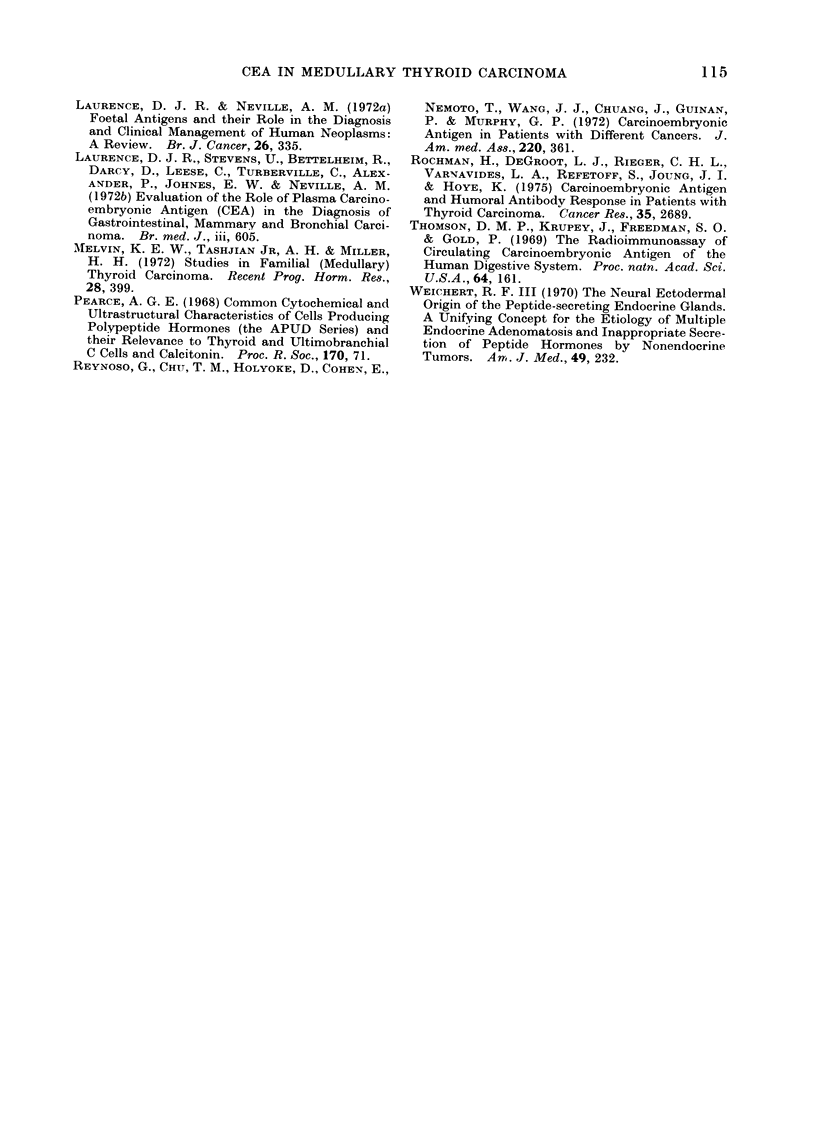# Association of medullary carcinoma of the thyroid with carcinoembryonic antigen.

**DOI:** 10.1038/bjc.1976.133

**Published:** 1976-08

**Authors:** N. Ishikawa, S. Hamada

## Abstract

**Images:**


*Br. J. Cancer* (1976) **34,** 111

### ASSOCIATION OF MEDULLARY CARCINOMA OF THE THYROID WITH CARCINOEMBRYONIC ANTIGEN

N. ISHIKAWA AND S. HAMADA*

*From the Department of Radiology, Kyoto University School of Medicine, and Radioisotope Research Centre, respectively, Kyoto University, Kyoto* 606, *Japan*

Received 5 March 1976 Accepted 12 April 1976

**Summary.—To investigate the association between medullary carcinoma of the thyroid (MCT) and carcinoembryonic antigen (CEA), we assayed 78 sera from patients with thyroid diseases for CEA, employing the radioimmunoassay of double antibody technique. All 13 sera from patients with MCT had high levels of CEA, ranging from 14 to 170 ng/ml. Increased serum CEA was noted even in cases of small, localized carcinoma. By contrast, serum CEA levels were normal (below 10 ng/ml) in all other histological types of thyroid carcinoma (33 cases), except for one case of papillary adenocarcinoma. In 32 patients with non-malignant thyroid diseases, with few exceptions serum CEA levels remained within the normal range. The elevated serum levels of CEA in MCT returned to normal after successful operation. Furthermore, very high tissue concentrations of CEA were demonstrated in MCT. The results indicate that CEA is actively produced by MCT, and that its measurement is useful in the diagnosis and management of the disease. It is suggested that the highly specific association of CEA with MCT may well be related to a defect of neural crest origin.**

MEDULLARY carcinoma of the thyroid (MCT) is known to secrete various bioactive substances such as calcitonin, serotonin, histaminase, etc., the unusual properties of which are exploited to establish early diagnosis in high-risk individuals and also to monitor the presence of metastatic disease following surgical treatment (Melvin, Tashjian and Miller, 1972). On the other hand, carcinoembryonic antigen (CEA), which was first found in colorectal carcinoma by Gold and Freedman (1965), is now considered to be a tumour-associated foetal antigen lacking specificity for particular tissues (Dykes and King, 1972; Laurence and Neville, 1972*a*). This paper describes the presence of CEA at high concentrations in sera and tumour tissues of MCT in contrast with other histological types of thyroid carcinoma.

### MATERIALS AND METHODS

Seventy-eight sera were obtained from patients with thyroid disease at the Thyroid Clinic of the Departments of Internal Medicine II and Radiology, Kyoto University Hospital. In all patients a definite diagnosis had been made by histological examination together with routine tests of thyroid function including scintigraphy. Blood samples were withdrawn by venepuncture, and the serum separated shortly thereafter was stored at −20°C until use.

### *Radioimmunoassay*

The assay method employed was the double antibody technique of nonequilibrium system, modified from the methods of Egan *et al.* (1972) and Laurence *et al.* (1972*b*). One hundred $\mu$l of serum sample was mixed with 100 $\mu$l of 5% normal rabbit serum in phosphate-buffered saline (PBS) at pH 7·4 and then with 100 $\mu$l of anti-CEA

* Reprints may be requested from: Satoshi Hamada, M.D., Radioisotope Research Centre, Kyoto University, Kyoto 606, Japan.

antiserum diluted 1 : 12,000. The mixture was incubated at 37°C for 2 h, and then 100 μl of $^{125}$I-CEA solution (60,000 ct/min) was added. After further incubation at 4°C overnight, 100 μl of 30% goat anti-rabbit IgG antiserum in PBS was also added, followed by incubation at 37°C for another 2 h. The mixture was centrifuged at 3000 rev/min for 30 min, and the radioactivity of the precipitate measured to estimate the amount of CEA it contained. The assay was run in duplicate, and was capable of determining CEA in amounts from 4 to 250 ng/ml with a standard deviation of ± 1 ng/ml for a mean value of 15 ng/ml. The upper limit of the normal range is 10 ng/ml. The values obtained (Y) were linearly proportional to those by the zirconyl phosphate gel assay (X), as expressed by a regression equation of $Y = 0{\cdot}99X + 1{\cdot}53$.

Purified CEA was prepared from perchloric acid extracts of colonic adenocarcinoma using sequential gel filtration on Sepharose 4B and Sephadex G-200 followed by preparative disc-gel electrophoresis (Hamada, Ishikawa and Yoshii, 1976). The preparations obtained were immunologically indistinguishable from that of Gold (kindly supplied by Dr P. Gold). The CEA was labelled with $^{125}$I by the chloramine T method. Specific anti-CEA antiserum was prepared after the method of Gold and Freedman (1965) by immunizing rabbits with perchloric acid extracts of the tumour, followed by absorption with normal sera and tissue extracts of colon, lung and liver.

### RESULTS

Serum levels and positivities of CEA in various types of thyroid carcinoma are shown in Fig. 1 and Table I. All 13 patients with medullary carcinoma of the thyroid (MCT) showed high levels of CEA, averaging 84 ng/ml. The values in 8 patients with metastases ranged from 14 to 170 ng/ml, averaging 97 ng/ml, while those in 5 patients without metastasis ranged from 48 to 125 ng/ml, averaging 73 ng/ml.

By contrast, all 33 patients with other histological types of thyroid carcinoma showed normal levels of CEA except for one case of papillary adenocarcinoma. In these diseases serum CEA remained within the normal range despite the presence of distant metastases.

Serum CEA levels and positivities in patients with nonmalignant thyroid diseases are shown in Fig. 2 and Table I. Only one of 9 patients with adenoma showed a CEA level of 19 ng/ml, and one of 7 patients with chronic thyroiditis showed a marginally elevated value (12 ng/ml). In all 14 patients with hyperthyroidism and 2 with thyroid cysts, the levels remained within the normal range.

Shown in Table II are changes in serum levels before and after surgical treatment of MCT. Although all CEA levels fell postoperatively, the values

Fig. 1.—Serum CEA levels in various types of thyroid carcinoma. In medullary carcinoma, open circles indicate localized disease, and closed circles metastatic disease.

TABLE I.—*Carcinoembryonic Antigen in Thyroid Diseases*

| | No. of patients | Positive | Metastasis + | Metastasis − | Metastasis Unknown |
|---|---|---|---|---|---|
| Medullary carcinoma | 13 | 13 | 9 | 4 | 0 |
| Papillary adenocarcinoma | 15 | 1 | 5 | 5 | 5 |
| Papillo-follicular adenocarcinoma | 8 | 0 | 3 | 3 | 2 |
| Follicular adenocarcinoma | 6 | 0 | 2 | 1 | 3 |
| Undifferentiated carcinoma | 4 | 0 | 4 | 0 | 0 |
| Adenoma | 9 | 1 | – | – | – |
| Cyst | 2 | 0 | – | – | – |
| Grave's disease | 14 | 0 | – | – | – |
| Hashimoto's thyroiditis | 7 | 1 | – | – | – |

FIG. 2.—Serum CEA levels in non-malignant diseases of the thyroid.

TABLE II.—*Preoperative and Postoperative Values for Serum CEA Levels in MCT*

| Case | Preoperative (ng/ml) | Postoperative* (ng/ml) | Mode of operation |
|---|---|---|---|
| 1 | 48 | 6 | Radical |
| 2 | 80 | 22 | Radical |
| 3 | 88 | 35 | Palliative |
| 4 | 145 | 60 | Palliative |
| 5 | 170 | 130 | Palliative |

* The specimens were taken 5–14 months after operation.

remained abnormally elevated in the cases of incomplete resection. After apparently radical operation, the serum CEA level returned to normal in one patient, but it was still slightly elevated in another patient requiring a follow-up.

Tissue concentrations of CEA were determined in 4 cases of MCT. The values ranged from 26 to 105 $\mu g/g$ wet tissue, averaging 64 $\mu g/g$, and represented 104–420 times that of hyperthyroidism.

### DISCUSSION

Although positive CEA findings initially appeared to be specific for malignancies of the gastrointestinal system (Thomson *et al.*, 1969), subsequent studies, employing more sensitive techniques of radioimmunoassay, showed that serum CEA increases in a variety of malignancies, ranging from pulmonary to genito-urinary systems and also in inflammatory bowel diseases (Dykes and King, 1972; Laurence *et al.*, 1972*a,b*; Booth *et al.*, 1973; Hansen *et al.*, 1974). It is now accepted, however, that the assay of serum CEA is most useful in the diagnosis of carcinoma of the gastrointestinal tract, pancreas and bronchus, because positive CEA findings are obtained in 70–90% of these diseases. It also appears valuable in the assessment of neuroblastoma and possibly of testicular and mammary neoplasms, since the CEA levels were elevated in all 6 patients with active neuroblastoma (Reynoso *et al.*, 1972) and in about half the patients with the other 2 diseases (Dykes and King, 1972; Laurence and Neville, 1972*a*).

However, its estimation is thought to be of little value in the diagnosis of tumours in other tissues.

Early sporadic studies on thyroid diseases showed that a positive CEA result was found in only one out of 9 patients with thyroid carcinoma but in none of 8 patients with adenoma or nodular goitre (Laurence *et al.*, 1972*b*; Reynoso *et al.*, 1972; Booth *et al.*, 1973). Quite recently Rochman *et al.* (1975) reported a more frequent elevation of CEA in their series including 37 patients with thyroid carcinoma, but the positivity was statistically significant only in cases with no previous history of childhood irradiation. In addition, no relationship was noted between levels of CEA and spread or histological appearance of the tumour.

However, the present study has revealed a highly specific association of raised CEA levels with MCT. The positivity obtained is much higher than in gastrointestinal malignancies and is comparable with that in neuroblastoma. Furthermore, the serum levels were in excess of 40 ng/ml in the majority (92%) of patients including those with localized disease. The levels are even greater than those in carcinomata of the gastrointestinal tract, bronchus and breast, in which serum CEA above this level is seen in only 12–13% of early and localized lesions and in 56–77% of distant metastases (Laurence *et al.*, 1972*b*; Booth *et al.*, 1973). It may be ascribed to the high content of CEA in the tumour cells and also to abundant blood supply to the endocrine organ. The results indicate that the assay of CEA is very useful in the diagnosis and follow-up of MCT.

In other histological types of thyroid carcinoma studied by us, serum CEA has remained mostly within the normal range, the findings being similar to those previously reported. It is likely, therefore, that the previous studies may include few or no cases of MCT, the incidence of which is 5–10% of thyroid carcinoma (Melvin *et al.*, 1972).

With few exceptions, high CEA levels were detected in MCT, which originates from the parafollicular cell. Interestingly enough, this cell is thought to be of neural crest origin and hence differs in its embryological derivation from the other parenchymal cells of the thyroid gland (Pearce, 1968; Weichert, 1970). Furthermore, it was reported that neuroblastoma, another neural crest tumour, showed a positive CEA finding in nearly all cases, and that the mean value was 4·5 ng/ml in the active disease as compared to the normal range of less than 2·5 ng/ml (Reynoso *et al.*, 1972). The lower level of CEA in the latter condition may well be due to a less rich blood supply to the tissue. It is therefore suggested that the highly specific association of raised CEA in the 2 neoplasmas may be related to a defect of neuroectodermal origin, although further studies should be performed with other neural crest tumours.

We are indebted to Dr Phil Gold, Montreal General Hospital, Montreal, for his generous supply of reference CEA and his kind advice in preparing CEA and anti-CEA antiserum, to Prof. Kanji Torizuka for his continuous encouragement, and to Dr Rikushi Morita for his kindness in supplying patient material.

### REFERENCES

BOOTH, S. N., KING, J. P. G., LEONARD, J. C. & DYKES, P. W. (1973) Serum Carcinoembryonic Antigen in Clinical Disorders. *Gut*, **14**, 794.

DYKES, P. W. & KING, J. (1972) Progress Report. Carcinoembryonic Antigen (CEA). *Gut*, **13**, 1000.

EGAN, M. L., LAUTENSCHLEGGER, J. T., COLIGAN, J. E. & TODD, C. W. (1972) Radioimmune Assay of Carcinoembryonic Antigen. *Immunochemistry*, **9**, 282.

GOLD, P. & FREEDMAN, S. O. (1965) Demonstration of Tumor-specific Antigens in Human Colonic Carcinomata by Immunological Tolerance and Absorption Techniques. *J. exp. Med.*, **121**, 439.

HAMADA, S., ISHIKAWA, N. & YOSHII, M. (1974) Purification of Carcinoembryonic Antigen (CEA) by Disc Gel Electrophoresis, 33rd Meeting Japan Cancer Ass., Sendai (Abst. 107).

HANSEN, H. J., SNYDER, J. J., MILLER, E., VANDEVOORDE, J. P., MILLER, O. N., HINES, L. R. & BURNS, J. J. (1974) Carcinoembryonic Antigen (CEA) Assay. A Laboratory Adjunct in the Diagnosis and Management of Cancer. *Hum. Path.*, **5**, 139.

LAURENCE, D. J. R. & NEVILLE, A. M. (1972*a*) Foetal Antigens and their Role in the Diagnosis and Clinical Management of Human Neoplasms: A Review. *Br. J. Cancer*, **26**, 335.

LAURENCE, D. J. R., STEVENS, U., BETTELHEIM, R., DARCY, D., LEESE, C., TURBERVILLE, C., ALEXANDER, P., JOHNES, E. W. & NEVILLE, A. M. (1972*b*) Evaluation of the Role of Plasma Carcinoembryonic Antigen (CEA) in the Diagnosis of Gastrointestinal, Mammary and Bronchial Carcinoma. *Br. med. J.*, iii, 605.

MELVIN, K. E. W., TASHJIAN JR, A. H. & MILLER, H. H. (1972) Studies in Familial (Medullary) Thyroid Carcinoma. *Recent Prog. Horm. Res.*, **28**, 399.

PEARCE, A. G. E. (1968) Common Cytochemical and Ultrastructural Characteristics of Cells Producing Polypeptide Hormones (the APUD Series) and their Relevance to Thyroid and Ultimobranchial C Cells and Calcitonin. *Proc. R. Soc.*, **170**, 71.

REYNOSO, G., CHU, T. M., HOLYOKE, D., COHEN, E., NEMOTO, T., WANG, J. J., CHUANG, J., GUINAN, P. & MURPHY, G. P. (1972) Carcinoembryonic Antigen in Patients with Different Cancers. *J. Am. med. Ass.*, **220**, 361.

ROCHMAN, H., DEGROOT, L. J., RIEGER, C. H. L., VARNAVIDES, L. A., REFETOFF, S., JOUNG, J. I. & HOYE, K. (1975) Carcinoembryonic Antigen and Humoral Antibody Response in Patients with Thyroid Carcinoma. *Cancer Res.*, **35**, 2689.

THOMSON, D. M. P., KRUPEY, J., FREEDMAN, S. O. & GOLD, P. (1969) The Radioimmunoassay of Circulating Carcinoembryonic Antigen of the Human Digestive System. *Proc. natn. Acad. Sci. U.S.A.*, **64**, 161.

WEICHERT, R. F. III (1970) The Neural Ectodermal Origin of the Peptide-secreting Endocrine Glands. A Unifying Concept for the Etiology of Multiple Endocrine Adenomatosis and Inappropriate Secretion of Peptide Hormones by Nonendocrine Tumors. *Am. J. Med.*, **49**, 232.